# LncNAP1L6 activates MMP pathway by stabilizing the m6A-modified NAP1L2 to promote malignant progression in prostate cancer

**DOI:** 10.1038/s41417-022-00537-3

**Published:** 2022-10-04

**Authors:** Yuxiao Zheng, Feng Qi, Lu Li, Bin Yu, Yifei Cheng, Minghui Ge, Chao Qin, Xiao Li

**Affiliations:** 1 Department of Urologic Surgery, Jiangsu Cancer Hospital & Jiangsu Institute of Cancer Research & Affiliated Cancer Hospital of Nanjing Medical University, Nanjing, China; 2 State Key Laboratory of Translational Medicine and Innovative Drug Development, Jiangsu Simcere Diagnostics Co., Ltd, Nanjing, China; 3 Nanjing Simcere Medical Laboratory Science Co., Ltd, Nanjing, China; 4 Department of Urology, The First Affiliated Hospital of Nanjing Medical University, Nanjing, China; 5 State Key Laboratory of Reproductive Medicine, Department of Urology, The First Affiliated Hospital of Nanjing Medical University, Nanjing, China; 6 Department of Scientific Research, Jiangsu Cancer Hospital & Jiangsu Institute of Cancer Research & Affiliated Cancer Hospital of Nanjing Medical University, Nanjing, China

**Keywords:** Tumour biomarkers, Oncogenes

## Abstract

Malignant progression such as bone metastasis, which is associated with pathologic fractures, pain and reduced survival frequently occurs in prostate cancer (PCa) patients at advanced stages. Accumulating evidence has supported that long non-coding RNAs (lncRNAs) participate in multiple biological processes. Nevertheless, the functions of most lncRNAs in PCa malignant progression remain largely unclear. Our current study is to elucidate the influence of lncRNA lncNAP1L6 on PCa malignant progression and uncover the possible regulatory mechanism. Firstly, RT-qPCR analysis was to detect lncNAP1L6 expression and suggested that lncNAP1L6 was markedly upregulated in PCa cells. Functional assays manifested that silencing of lncNAP1L6 hampered cell migration, invasion, and epithelial-mesenchymal transition (EMT) while overexpression of lncNAP1L6 exacerbated cell migration, invasion and EMT. In addition, mechanism assays were to determine the latent regulatory mechanism of lncNAP1L6. It turned out that METTL14/METTL3 complex mediated m6A methylation of NAP1L2 mRNA. Besides, lncNAP1L6 recruited HNRNPC to m6A-modified NAP1L2, leading to stabilization of NAP1L2 mRNA. Moreover, NAP1L6 interacted with YY1 to promote the transcription of MMP2 and MMP9 and activate MMP signaling pathway. In summary, lncNAP1L6 was identified as an oncogene in PCa, which revealed that lncNAP1L6 might be used as potential therapeutic target in PCa.

## Introduction

Prostate cancer (PCa) which ranks second among the most frequently diagnosed cancers in men is a slow-growing malignancy [1]. The incidence and mortality of PCa in China has increased instantly due to the growth of the elderly population, westernized diet habits, widespread environment contamination and so on [2]. Bone metastasis, as a representative late-stage event of malignant tumor progression of most common cancers, such as breast cancer, prostate cancer and so on. As a result, pain, increased risk of fracture and hypercalcemia may occur [3]. Moreover, tumor and bone interact with each other, where tumor-secreted factors stimulate bone cells, which in turn release growth factors and cytokines that act back on the tumor cells [4]. Despite recent advances in therapeutic approaches, malignant progression remains incurable and multiple complications may occur, including hypercalcemia, pathological fractures, bone pain and so on [5]. Hence, exploring the mechanism under malignant tumor progression in PCa is of great significance to the therapy for PCa.

Long non-coding RNAs (lncRNAs) are commonly defined as novel class of non-coding RNAs having lengths of 200 nucleotides. It is considered that lncRNAs are one of the emerging regulators in diverse cellular processes in the development of cancers [6]. For example, Peng et al. have revealed that lncRNA PSMA3-AS1 play oncogenic roles in colorectal cancer [7]. Chen et al. have demonstrated that HOXD-AS1 aggravates the progression of cervical cancer via sequestering miR-877-3p and upregulating FGF2 [8]. Recent studies have also manifested that lncRNAs exert important roles in the pathogenesis of PCa [9]. For instance, Li et al. have underlined that lncRNA BLACAT1 suppresses the development of PCa by serving as a sponge for miR-361 [10]. Hu et al. have implied that lncRNA HCP5 accelerates cell proliferation in PCa via miR-4656/CEMIP axis [11]. He et al. have proposed that lncRNA CCAT2 contributes to cell proliferation and invasion in PCa through modulation of Wnt/β-catenin signaling pathway [12]. LncNAP1L6 has been mentioned to act as an oncogene in PCa. It was reported that LncNAP1L6 expression was associated with prognosis in PCa. Besides, LncNAP1L6 can promoted cell migration and expression of Vimentin and beta-catenin, and also inhibits the expression of E-cadherin in PCa [13, 14]. Nevertheless, whether lncNAP1L6 is associated with malignant progression of PCa remains elusive. The purpose of this study is to disclose the function of lncNAP1L6 on malignant progression in PCa and probe into the underlying regulatory mechanism.

## Materials and methods

### Cell culture

Human PCa cell lines (22RV1, PC3, C4-2B, VCaP and DU145) and normal human prostatic epithelial cell line (RWPE-1) were purchased from American Type Culture Collection (ATCC, Mannassas, VA, USA) and maintained in RPMI-1640 Medium (CD-02168-ML, GIBCO, USA) added with 10% fetal bovine serum (FBS, 10270-106, GIBCO, USA) and 100 U/ml streptomycin under the condition of 37°C and 5% CO2.

### Cell transfection

The shRNAs targeting lncNAP1L6, METTL3, METTL14, HNRNPC, NAP1L2 or YY1 and control shRNA plasmid were all obtained from Ribobio (Guangzhou, China). Additionally, pcDNA3.1/lncNAP1L6, pcDNA3.1/METTL14, pcDNA3.1/HNRNPC, pcDNA3.1/NAP1L2 and NC vector were available from GenePharma Company. Lipofectamine 2000 (XFSJ16444, GIBCO, USA) was used for cell transfection.

### Quantitative real-time PCR (RT-qPCR)

After total cellular RNA was collected, Reverse Transcription Kit (11141ES10, Takara, Japan) was employed to convert total RNA into cDNA. By using a SYBR Green Real-Time PCR Kit (QR0100-1KT, Sigma-Aldrich, USA), RT‑qPCR was performed. β-actin was viewed as an internal control and the relative gene expression was determined by the 2 − ΔΔCt method. The assay was independently carried out in triplicate.

### Wound healing assay

After collected, the cells were transferred to serum-free medium for incubation and a straight scratch wound was made using a pipette tip when cells reached 80% confluence. After incubation for another 24 h, scratches were monitored and photographed. The experiment was independently conducted in triplicate.

### Transwell assay

In detail, the upper chambers of Transwell inserts (Corning Incorporated, Corning, NY) were used to accommodate PCa cells. Transwell inserts covered by Matrigel was used for invasion assay, whereas transwell inserts without Matrigel membrane was applied for migration detection. Crystal violet was used to dye cells for counting 24 h later. The assay was independently carried out in triplicate.

### RNA immunoprecipitation (RIP)

With the application of Imprint® RNA Immunoprecipitation Kit (RIP-12RXN, Sigma-Aldrich, USA), RIP assay was implemented. PCa cells were collected and then lysed in RIPA lysis buffer (RIPA2-11-527, TBD, China). Subsequently, lysates were immunoprecipitated with anti-METTL14 (ab220030, Abcam), anti-HNRNPC (Abcam) or anti-IgG (Abcam). Finally, the RNA precipitates were extracted and then analyzed by RT-qPCR. The assay was independently implemented in triplicate.

### MeRIP assay

A total of 3 µg RNA and m6A spike-in control mixture suspended in IP buffer was incubated at 4 °C for 2 h with 2 µg anti-m6A antibody. After being washed and further incubated at 4 °C for 2 h with Dynabeads M-280 Sheep Anti-Rabbit IgG (Thermo Scientific), the beads were washed with IP buffer for 3 times and with wash buffer for 2 times. Eventually, the enriched RNA was eluted with elution buffer and purified using the RNeasy Mini Kit (Qiagen). The assay was independently implemented in triplicate.

### Chromatin immunoprecipitation (ChIP)

PC3 cells were fixed in 1% formaldehyde for 30 min at room temperature. Sonication was used to cut the DNA to an average size. For chromatin immunoprecipitation, anti-YY1 antibody was utilized and anti-lgG was used as the negative control. RT-qPCR was conducted to quantify the purified chromatin. The assay was independently implemented in triplicate.

### RNA pull-down assays and mass spectrometry

Biotinylated full-length of lnNAP1L6 or NAP1L2, antisense lnNAP1L6 and antisense NAP1L2 were synthesized. RNA pull-down assays were determined via a PierceTM Magnetic RNA-Protein Pull-Down Kit (Thermo Fisher, USA). The sequences were hatched with cell lysates at room temperature for 30 min, and then added with streptavidin magnetic beads (Thermo, USA) at 4 °C overnight. The proteins were separated by electrophoresis and visualized. The different bands between sense and antisense lncNAP1L6 were assessed via mass spectrometry. The assay was independently implemented in triplicate.

### Co-Immunoprecipitation (Co-IP) assay

The prepared cell lysates were collected from the treated cells in IP lysis buffer, and then cultured with indicated antibodies and control IgG antibody overnight at 4 °C. After mixed with beads, samples were washed in IP lysis buffer and assayed by western blot. The assay was independently carried out in triplicate.

### Luciferase reporter assay

The NAP1L2 sequence containing the binding sites (wild type or mutant type) was constructed into the pGL3 vector (Promega, Madison, WI) and co-transfected along with pcDNA3.1/METTL14, pcDNA3.1/HNRNPC or the empty vector into PC3 cells. Similarly, the luciferase activity of MMP2 or MMP9 promoter was also detected after co-transfection with pcDNA3.1/YY1 or the empty vector. After 48 h, the luciferase activity was measured with the employment of the Dual-Luciferase Reporter Gene Assay Kit (Yeasen, Shanghai, China) was applied to detect the luciferase activity. The assay was independently carried out in triplicate.

### Western blot analysis

After being separated by SDS-PAGE (P1200, Solarbio, China), total proteins were transferred onto PVDF membranes (Millipore, Billerica, MA, USA). Afterwards, membranes were incubated by primary antibodies at 4 °C overnight. Then the blots were then incubated with secondary antibody for 1 h at dark room after washed with PBS for three times. The primary antibodies were as follows: ZO-1 (ab276131, Abcam), E-cadherin (ab40772, Abcam), N-cadherin (ab76011, Abcam), Vimentin (ab92547, Abcam), Fibronectin, NAP1L2 (K21628-RNX, Biolab), HNRNPC, METTL14 (ab220030, Abcam), YY1 (ab109237, Abcam), MMP2 (ab92536, Abcam), MMP9 (ab76003, Abcam) and β-actin (ab8226, Abcam). Protein quantification was conducted by Immobilon Western Chemiluminescent HRP Substrate (Merck Millipore, Billerican MA, USA). The assay was independently carried out in triplicate.

### Dot blot assay

Briefly, 40 ng of denatured mRNA was spotted onto Hybond N + membrane (GE Healthcare), UV cross-linked, blocked with SuperBlock Blocking Buffer (Thermo Scientific) and probed with rabbit anti-m6A antibody (1:250, Synaptic Systems). Blots were visualized with enhanced chemiluminescence and quantified using Image Studio software (LI-COR Biotechnology). The assay was independently carried out in triplicate.

### Statistical analysis

SPSS version 16.0 (IBM, Chicago, IL) was used to analyze data. The data are displayed as the means ± SD. All experiments were conducted in triplicate. Unpaired two-tailed Student’s *t*-test or one-way ANOVA was used to analyze the differences between two or more groups. *P*-value < 0.05 was thought to be statistically significant.

## Results

### lncNAP1L6 promoted cell migration, invasion and EMT in PCa

**Fig. 1:**
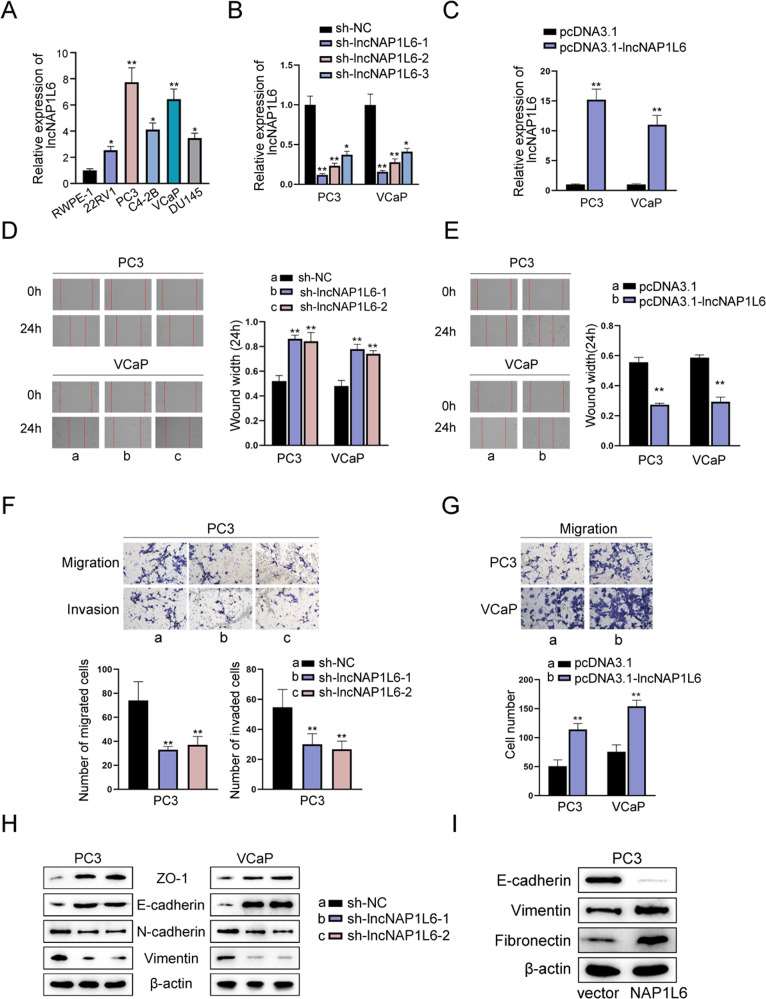
lncNAP1L6 promoted cell migration, invasion and EMT in PCa. **A** RT-qPCR examine lncNAP1L6 expression in PCa cell lines (22RV1), PCa bone metastasis cell lines (PC3, C4-2B and VCaP) and PCa brain metastasis cell line (DU145). **B** LncNAP1L6 was knocked down in PC3 and VCaP cells. **C** LncNAP1L6 was overexpressed in PC3 and VCaP cells. **D**, **E** Wound healing assays evaluated the migration of PCa cells. **F** Transwell assays assessed cell migration and invasion when lncNAP1L6 was downregulated. **G** Cell migration was observed through transwell assays when lncNAP1L6 was upregulated. **H** Western blot analyzed the protein levels of ZO-1, E-cadherin, N-cadherin and Vimentin after lncNAP1L6 was silenced. **I** Western blot analyzed the protein levels of E-cadherin, Vimentin and Fibronectin after lncNAP1L6 was overexpressed. *P < 0.05, **P < 0.01.

Through RT-qPCR analysis, we discovered that lncNAP1L6 was obviously overexpressed in PCa cell lines (22RV1), PCa bone metastasis cell lines (PC3, C4-2B and VCaP) and PCa brain metastasis cell line (DU145) in comparison with normal cell line (RWPE-1) (Fig. 1A). Moreover, PC3 and VCaP cells were selected for subsequent studies because of the highest expression. To assess the role of lncNAP1L6 in PCa, we firstly knocked down lncNAP1L6 and overexpressed lncNAP1L6 in PC3 and VCaP cells respectively. As demonstrated in Fig. 1B, C, the interference efficiency and overexpression efficiency of lncNAP1L6 were examined. Through wound healing assays, we observed that wound width was increased when lncNAP1L6 was downregulated while wound width was decreased when lncNAP1L6 was upregulated (Fig. 1D, E), indicating that silencing of lncNAP1L6 impeded the migration of PCa cells and overexpression of lncNAP1L6 accelerated the migration of PCa cells. In addition, transwell migration and invasion assays also implied that interference of lncNAP1L6 inhibited cell migration and invasion in PCa while lncNAP1L6 upregulation aggravated cell migration in PCa (Fig. 1F, G). Western blot analysis also uncovered that the protein levels of ZO-1 and E-cadherin were enhanced while the protein levels of N-cadherin and Vimentin were reduced after lncNAP1L6 was inhibited (Fig. 1H). Conversely, lncNAP1L6 overexpression decreased the protein level of E-cadherin and increased the protein levels of Vimentin and Fibronectin (Fig. 1I). To be concluded, lncNAP1L6 played the promoting role in cell migration, invasion and EMT process in PCa. **P < 0.01.

### LncNAP1L6 elevates the expression of METTL3/METTL14-induced m6A methylated NAP1L2

**Fig. 2:**
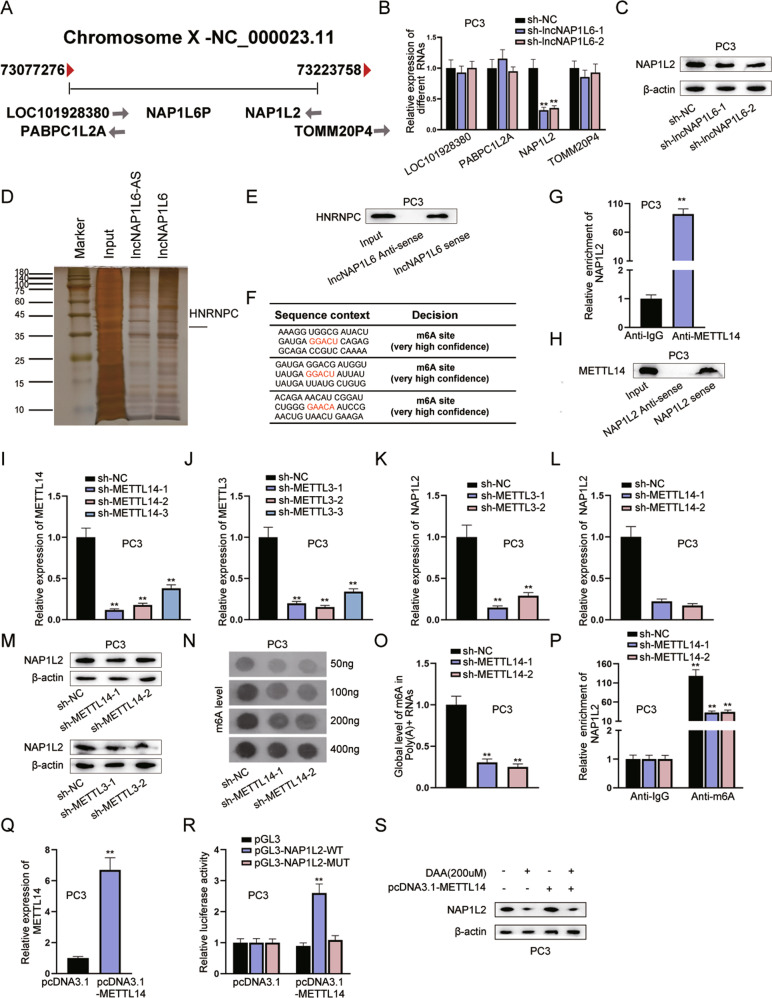
LncNAP1L6 positively regulates METTL3/METTL14-mediated m6A-modified NAP1L2. **A** NCBI database predicted nearby genes of NAP1L6. **B** LOC101928380, PABPC1L2A, NAP1L2 and TOMM20P4 mRNA levels were tested after lncNAP1L6 was depleted. **C** Western blot analyzed the protein level of NAP1L2 when lncNAP1L6 was downregulated. **D** HNRNPC was discovered to interact with lncNAP1L6 through RNA pull down and mass spectrometry. **E** RNA pull-down assay verified the interaction between lncNAP1L6 and HNRNPC. **F** SRAMP website validated that NAP1L2 was modified by m6A methylation. **G**, **H** RIP and RNA pull-down assays verified the interaction between METTL14 and NAP1L2. **I**–**M** NAP1L2 expression was examined when METTL14 or METTL3 was downregulated. **N** Dot blot assay tested m6A level after METTL14 was knocked down. **O** Global m6A levels were detected after METTL14 was silenced. **P** MeRIP assay uncovered the abundance of NAP1L2 in m6A antibody when METTL14 was downregulated. **Q** The overexpression efficiency of METTL14 was tested. **R** Luciferase reporter assay examined the luciferase activity of NAP1L2-WT and NAP1L2-MUT when METTL14 was overexpressed. **S** NAP1L2 protein level was tested in different groups. **P < 0.01.

As exhibited in Fig. 2A, through NCBI database (https://www.ncbi.nlm.nih.gov/), we observed that LOC101928380, PABPC1L2A, NAP1L2 and TOMM20P4 are nearby genes of NAP1L6. Further, RT-qPCR analysis uncovered that among these four genes, NAP1L2 expression was significantly lessened after transfection of sh-lncNAP1L6#1/2 plasmids (Fig. 2B). Therefore, NAP1L2 was chosen for subsequent studies as potential target gene. Similarly, the protein level of NAP1L2 was also cut down after lncNAP1L6 was silenced (Fig. 2C). To explore the regulatory mechanism between lncNAP1L6 and NAP1L2, we conducted RNA pull-down assay followed by silver staining. As displayed in Fig. 2D, a protein band specifically presented in lncNAP1L6 was located at ~42 kDa and then HNRNPC was selected by sequence analysis via mass spectrometry. The interaction between lncNAP1L6 and HNRNPC was also verified by RNA pull-down assay (Fig. 2E). As reported, HNRNPC is a m6A RNA methylation regulator which is able to identify the m6A sites [15]. At the same time, previous studies have confirmed that METTL3 and METTL14 are highly expressed in PCa cells [16]. The association between METTL3 and NAP1L2 expression has also been increased in Fig. S1A. The evidence that METTL14 is positively associated with NAP1L2 has been added in Fig. S1B. The data are derived from the analysis of PRAD tissue in the GEPIA database (http://gepia.cancer-pku.cn/detail.php). We used pearson correlation coefficient to measure the correlation, and the *P*-value < 0.05 indicated that the correlation between the expression of NAP1L2 and METTL3/METTL4 is statistically significant. Following, whether METTL14-mediated m6A methylation of NAP1L2 was investigated. Through SRAMP website (http://www.cuilab.cn/sramp/), there was a m6A site with high confidence on the sequence of NAP1L2 (Fig. 2F). In addition, RIP and RNA pull-down assay validated the interaction between METTL3 and NAP1L2 mRNA (Fig. 2G, H). Liu et al. have reported that METTL3/METTL14 m(6)A RNA methyltransferase complex plays crucial regulatory roles in m6A methylation modification [17]. RT-qPCR and western blot analysis suggested that NAP1L2 mRNA levels and protein levels were both cut down after METTL14 or METTL3 was depleted (Fig. 2I–M), indicating that METTL14 and METTL3 positively regulated NAP1L2 expression. METTL14 inhibition led to the decrease on the m6A levels with the increasing concentrations of total RNA levels (50 ng, 100 ng, 200 ng, 400 ng) (Fig. 2N). With the application of RNA total m6A quantification (P-9008-48-E, Epigentek, USA), we discovered that global m6A levels were decresed when METTL14 was downregulated (Fig. 2O). Further, MeRIP attested that METTL14 deficiency reduced the enrichment of NAP1L2 in m6A antibody (Fig. 2P). Luciferase reporter assay also confirmed that METTL14 overexpression enhanced the luciferase activity of PGL3-NAP1L2-WT group (Fig. 2R). Additionally, western blot analyzed that after treated with 3-deazaadenosine (DAA), an RNA methylation inhibitor, NAP1L2 protein level was decreased while this influence was rescued by METTL14 upregulation (Fig. 2S). We performed the meRIP assay and found that the total m6A level in PC3 cells was significantly reduced after METTL3 interference (Fig. S1C); and we found that the m6A level of NAP1L2 mRNA was reduced after METTL3 interference (Fig. S1D). In summary, m6A methylation of NAP1L2 mRNA was mediated by METTL14.

### LncNAP1L6 recruits HNRNPC protein to stabilize m6A-modified NAP1L2 mRNA

**Fig. 3:**
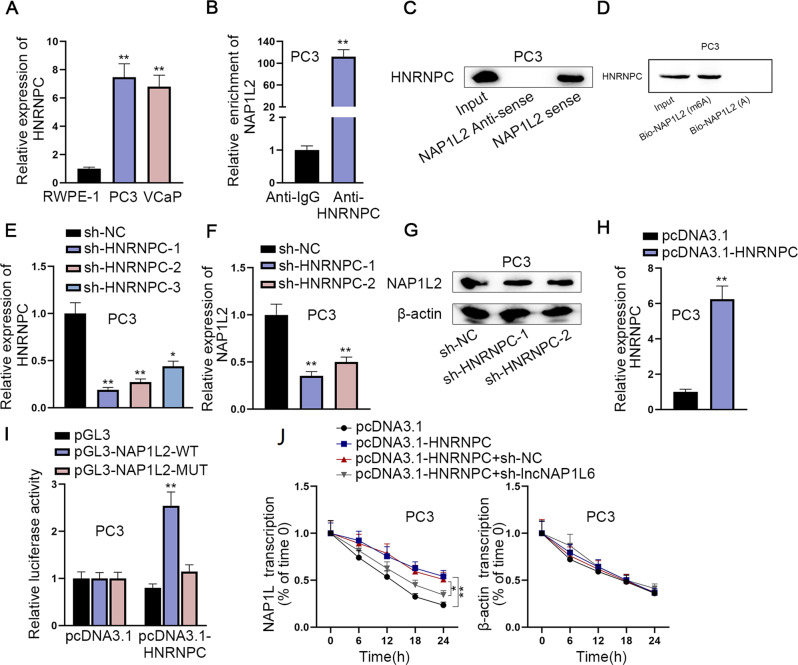
LncNAP1L6 recruits HNRNPC protein to stabilize m6A-modified NAP1L2 mRNA. **A** HNRNPC expression was detected in RWPE-1, PC3 and VCaP cells. **B**, **C** The interaction between HNRNPC and NAP1L2 was confirmed by RIP and RNA pull down assays. **D** RNA pull-down assays demonstrate that HNRNPC binds to m6A-modified NAP1L2. **E** HNRNPC expression was tested after HNRNPC was silenced. **F**, **G** NAP1L2 expression was tested after HNRNPC was silenced. **H** HNRNPC expression was elevated in PC3 cells. **I** Luciferase reporter assay examined the luciferase activity of NAP1L2-WT and NAP1L2-MUT after HNRNPC was overexpressed. **J** The stability of NAP1L2 mRNA was examined in the pcDNA3.1 group, pcDNA3.1-HNRNPC group, pcDNA3.1-HNRNPC + sh-NC group and pcDNA3.1-HNRNPC + sh-lncNAP1L6 group. **P < 0.01.

Through RT-qPCR analysis, HNRNPC was discovered to be overexpressed in PC3 and VCaP cell lines compared with RWPE-1 cell line (Fig. 3A). The strong affinity of HNRNPC with NAP1L2 was also testified by RIP and RNA pull-down assays (Fig. 3B, C). We performed RNA pull-down assays in Fig. 3D to demonstrate that HNRNPC binds to m6A-modified NAP1L2. Additionally, RT-qPCR and western blot analysis implied that downregulation of HNRNPC cut down the expression of NAP1L2 (Fig. 3D–F). Luciferase reporter assay further proved that overexpression of HNRNPC enhanced the luciferase activity of pGL3-NAP1L2-WT group (Fig. 3G, H). Finally, after treated with 50 mM α-amanitin, HNRNPC overexpression enhanced the stability of NAP1L2 mRNA while this effect was restored by lncNAP1L6 deficiency (Fig. 3I). In summary, HNRNPC identified m6A-modified NAP1L2 to maintain the stability of NAP1L2 mRNA.

### NAP1L2 contributes to the migration and EMT process of PCa cells

**Fig. 4:**
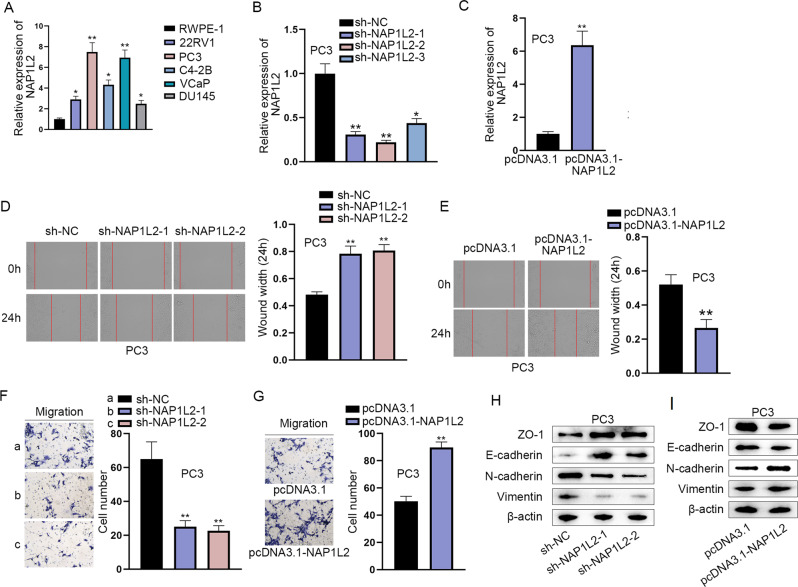
NAP1L2 contributes to the migration, invasion and EMT process of PCa cells. **A** RT-qPCR examine NAP1L2 expression in PCa cell lines (22RV1), PCa bone metastasis cell lines (PC3, C4-2B and VCaP) and PCa brain metastasis cell line (DU145). *P < 0.05, **P < 0.01. **B** NAP1L2 was knocked down in PC3 cells. **C** NAP1L2 was overexpressed in PC3 cells. **D**–**G** Cell migration was observed through wound healing assays when NAP1L2 was downregulated or upregulated. **H**, **I** Western blot analyzed the protein levels of ZO-1, E-cadherin, N-cadherin and Vimentin after NAP1L2 was silenced or overexpressed. **P < 0.01.

From RT-qPCR analysis, we found that NAP1L2 exhibited higher expression in PCa cell lines (22RV1), PCa bone metastasis cell lines (PC3, C4-2B and VCaP) and PCa brain metastasis cell line (DU145) in comparison with normal cell line (RWPE-1). Moreover, NAP1L2 expression was the most elevated in PCa bone metastasis cell lines (PC3 and VCaP) (Fig. 4A). Summarily, NAP1L2 exhibited high expression in PCa cells, especially in bone metastasis cells. To detect the role of NAP1L2 in the malignant phenotypes of PCa cells, functional experiments were implemented. Firstly, the interference and overexpression efficiency of NAP1L2 was examined after transfection of sh-NAP1L2-1/2/3 plasmids and pcDNA3.1-NAP1L2 plasmids (Fig. 4B, C). The experimental results of wound healing and transwell migration assays exposed that reduction of NAP1L2 attenuated the migratory ability of PC3 cells and NAP1L2 upregulation strengthened the migratory capacity (Fig. 4D–G). Western blot analyzed that NAP1L2 knockdown enhanced the protein levels of ZO-1 and E-cadherin while lessened the protein levels of N-cadherin and Vimentin (Fig. 4H). On the contrary, NAP1L2 overexpression lessened the protein levels of ZO-1 and E-cadherin while enhanced the protein levels of N-cadherin and Vimentin (Fig. 4I). Taken together, NAP1L2 played the promoting role in cell migration and EMT in PCa.

### NAP1L2/YY1 complex promotes the transcription of MMP2 and MMP9

**Fig. 5:**
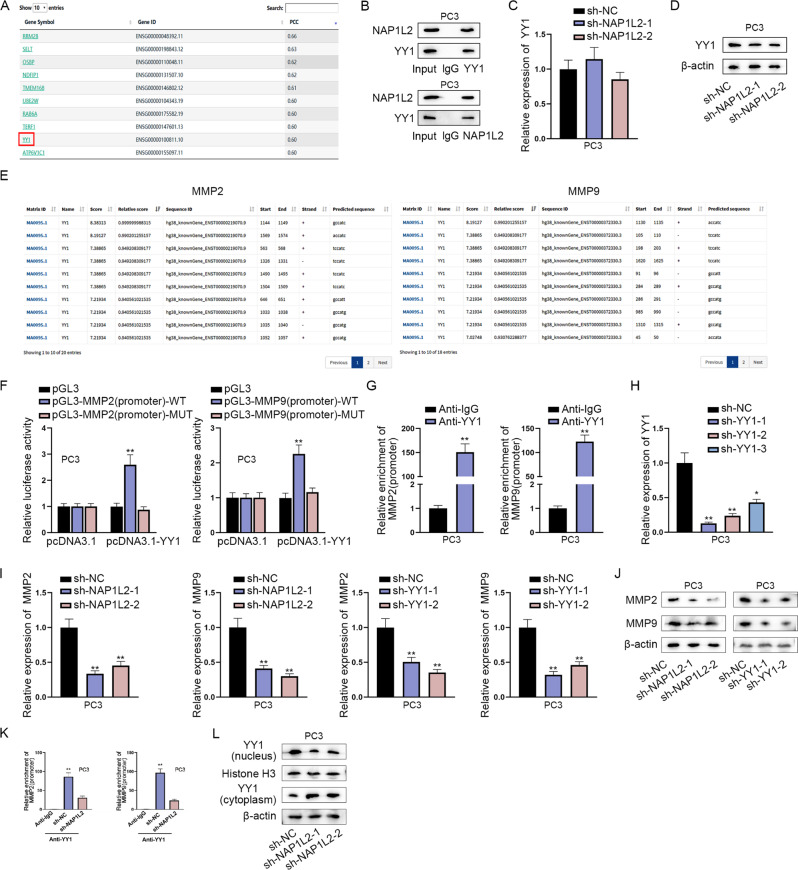
NAP1L2/YY1 complex promotes the transcription of MMP2 and MMP9. **A** GEPIA database predicted co-expression genes of NAP1L2 in PCa. **B** Co-IP assay validated the binding between NAP1L2 and YY1. **C**, **D** YY1 expression was tested after NAP1L2 was silenced. **E** JASPAR database predicted the binding sites between YY1 and MMP2 promoter and MMP9 promoter. **F**, **G** Luciferase reporter assays and ChIP assays testified the interaction between YY1 and MMP2 promoter or MMP9 promoter. **H** YY1 was knocked down in PC3 cells. **I**, **J** MMP2 and MMP9 expression were detected when NAP1L2 or YY1 was downregulated. **K** ChIP assay examined the enrichment of MMP2 and MMP9 promoter in YY1 antibody when NAP1L2 was downregulated. **L** Western blot analyzed the protein levels of YY1 in nucleus and cytoplasm when NAP1L2 was downregulated. **P < 0.01.

We used the ‘Similar Genes’ module in the GEPIA database. We set ‘Gene’ as NAP1L2, set ‘TCGA tumor’ as prostate cancer tumor, set ‘TCGA normal’ as prostate cancer normal, and then performed co-expression gene analysis. We selected the top 20 genes in the results and focused on genes with PCC values ≥ 0.6, as this suggested that the two genes were highly correlated. The top 20 genes were showed in Table S1. Among these possible genes, YY1 was selected for that YY1 is an important regulator in tumor metastasis (Fig. 5A) [18]. Co-IP assay proved that NAP1L2 had a binding with YY1 (Fig. 5B). However, RT-qPCR and western blot analyzed that silencing of NAP1L2 exerted no influence on YY1 expression (Fig. 5C, D). Intriguingly, as a common transcription factor, YY1 has been reported to promote the transcription of MMP2 [19]. MMP2 and MMP9 signaling pathway is identified as a vital participator in PCa cell migration, invasion and EMT [20]. Following, we speculated that NAP1L2/YY1 complex bound to MMP2 promoter and MMP9 promoter to promote the transcription of MMP2 and MMP9. Through JASPAR database (http://jaspar.genereg.net/), the potential binding sites between YY1 and MMP2 or MMP9 were displayed (Fig. 5E, Table S2, Table S3). Luciferase reporter assays elucidated that YY1 induced the increase on the luciferase activities of pGL3-MMP2 (promoter)-WT group and pGL3-MMP9 (promoter)-WT group (Fig. 5F). Furthermore, ChIP assays clarified that MMP2 and MMP9 promoter were abundant in YY1 antibody (Fig. 5G). To knock down YY1, sh-YY1-1/2/3 plasmids were transfected into PC3 cells (Fig. 5H). Moreover, MMP2 and MMP9 expression was decreased when NAP1L2 or YY1 was downregulated (Fig. 5I, J). Additionally, ChIP assays demonstrated that after NAP1L2 was silenced, the abundance of MMP2 promoter and MMP9 promoter in YY1 antibody was reduced (Fig. 5K). Meanwhile, western blot analysis suggested that when NAP1L2 was depleted, YY1 expression in the nucleus was decreased and in the cytoplasm was increased (Fig. 5L), which implied that NAP1L2 interacted with YY1 and promoted YY1 nuclear translocation to promote the transcription of MMP2 and MMP9. Collectively, MMP2 and MMP9 transcription were induced by NAP1L2/YY1 complex.

**Fig. 6:**
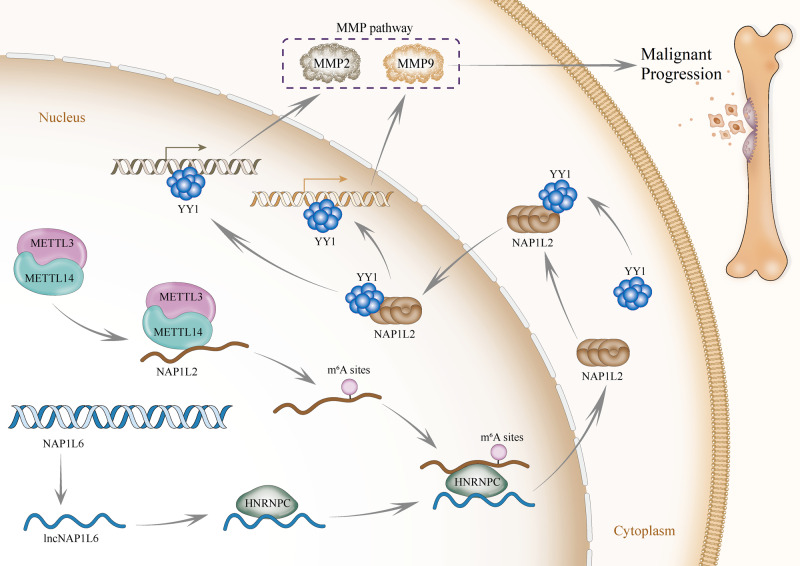
Schematic representation. Schematic representation of the proposed mechanisms.

In conclusion, METTL14/METTL3 complex induces m6A methylation of NAP1L2. Moreover, lncNAP1L6 recruites HNRNPC protein to m6A-modified NAP1L2 and stabilize NAP1L2 mRNA. Furthermore, NAP1L2 interacts with YY1 to aggravate the contribution of YY1 to the transcription of MMP2 and MMP9. Therefore, we can summary that, lncNAP1L6 activated MMP pathway facilitates PCa cell migration, invasion, EMT process and malignant progression (Fig. 6).

## Discussion

Malignant progression of PCa, such as bone metastasis, represents a significant medical problem which causes significant morbidity and mortality [21]. LncRNAs have been widely investigated in bone metastasis in PCa. For example, Zhu et al. have exposed that lncRNA H19 plays suppressive roles in PCa bone metastasis via sequestering miR-675 and targeting TGFB1 [22]. Hu et al. have revealed that NORAD/miR-541-3p/PKM1 axis induces bone metastasis of PCa [23]. LncNAP1L6 has been reported to contribute to PCa progression and be associated with poor prognosis [13]. Consistent with these findings, lncNAP1L6 expression was high in PCa cells. Moreover, lncNAP1L6 deficiency repressed cell migration, invasion and EMT process in PCa.

METTL3 and METTL14 are core m6A writers containing methyltransferase domains in RNA modification [24]. Through our investigation, NAP1L2 was discovered to be a nearby gene of lncNAP1L6 and positively regulated by lncNAP1L6. Furthermore, m6A methylation of NAP1L2 was mediated by METTL14. In addition, NAP1L2 was notably overexpressed in PCa cells and facilitated the migration and EMT process of PCa cells.

HNRNPC has been identified as an m6A methylation reader which can affect mRNA in an m6A-dependent manner [15, 25]. Our experimental results confirmed that lncNAP1L6 bound to HNRNPC protein. Also, HNRNPC identified m6A sites of NAP1L2 and stabilized NAP1L2 mRNA. The enhanced stability of NAP1L2 mRNA induced by HNRNPC overexpression was restored by lncNAP1L6 knockdown.

As a common transcription factor, YY1 has been reported to widely influence gene transcription in various kinds of cancers [26–28]. Besides, YY1 has been considered to be an inducer of cancer metastasis [18]. YY1 has been also uncovered to be concerned with the pathogenesis of PCa [29]. In our study, we demonstrated that NAP1L2 had a strong binding with YY1 and promoted YY1 nuclear translocation. At the same time, YY1 interacted with MMP2 promoter and MMP9 promoter to activate the transcription of MMP2 and MMP9.

In summary, lncNAP1L6 was upregulated in PCa cells and promoted PCa cell migration, invasion, EMT process and malignant progression. From the perspective of mechanism, METTL14/METTL3 complex promoted m6A methylation of NAP1L2. Moreover, lncNAP1L6 recruited HNRNPC protein to stabilize m6A-modified NAP1L2 mRNA. Furthermore, NAP1L2 cooperated with YY1 to promote the transcription of MMP2 and MMP9, which promoted tumor metastasis.

## Supplementary information

Supplementary File 1

Supplementary File 2

Supplementary Legends

Supplementary tables

Figure S1

## Data Availability

The data used to support the findings of this study are available from the corresponding author upon request.
