# Supplementary material for: LncNAP1L6 activates MMP pathway by stabilizing the m6A-modified NAP1L2 to promote malignant progression in prostate cancer
Source: Cancer Gene Ther. 2022 Oct 4;30(1):209–18. doi: 10.1038/s41417-022-00537-3 (PMC9842505; doi:10.1038/s41417-022-00537-3)
Supplement: Supplementary file 1 [file 41417_2022_537_MOESM1_ESM.docx]

Plasmid sequences in luciferase reporter assays：

pGL3-NAP1L2-WT:

5’-AAGTGATAGCTGCGTAATCATACTGCGGCACCGTTTTTTTCTTGCAGCAGTAGCTGCT

TGCGGAGGAGGTCTGCCCACTGCAGCTCTCTGCAGTCTCCGGCTCTCTCCTGCAGGATCGGTCAACGCAGCCGTCGCCGCCCTCTGCACCCAGCCCAGGTCGCCACTGCTTCAGTCCGGTTCTCAAAGCCTCAGCACCATCTTTTATCCCCGAGCAGCCTGGATCGTCGTTCCCTCAGTCCGGACGCCACTGCTAGGTCCGACCACCGCCGCTTCTGATATTTCGGTGAGTCTTTTCCTGTGGAGGTTTGGTCTCCCGATCTCTGTGGTAGCCACCTTAGGCGTGTACGGTCCTTTGAAAAATGGCCGAGTCAGAGAACCGCAAGGAGCTGTCAGAATCCAGTCAAGAAGAGGCTGGTAATCAGATAATGGTGGAAGGGCTCGGGGAACATCTGGAGCGCGGTGAAGATGCCGCTGCTGGGCTTGGAGACGATGGGAAGTGCGGTGAAGAAGCTGCCGCTGGGCTTGGGGAAGAAGGGGAAAACGGTGAAGATACTGCTGCTGGGTCCGGGGAAGATGGGAAAAAAGGTGGCGATACTGATGAGGACTCAGAGGCAGACCGTCCAAAAGGACTTATCGGTTATGTTTTAGATACAGACTTTGTTGAAAGTCTACCTGTGAAAGTTAAGTACCGTGTGTTAGCCCTTAAAAAGCTTCAAACTAGAGCGGCCAATTTAGAATCCAAATTCCTGAGGGAATTTCATGACATTGAAAGAAAGTTTGCTGAAATGTACCAACCCTTACTGGAAAAAAGACGTCAGATCATCAATGCAATCTATGAACCTACAGAAGAGGAATGTGAATATAAATCAGACTCTGAGGACTGTGATGATGAGGAAATGTGTCATGAAGAGATGTATGGTAATGAGGAGGGTATGGTACATGAATATGTGGATGAGGACGATGGTTATGAGGACTATTATTATGATTATGCTGTGGAAGAGGAGGAGGAGGAGGAGGAGGAGGACGACATTGAGGCTACTGGAGAAGAGAATAAAGAAGAGGAGGATCCTAAGGGAATTCCTGATTTTTGGCTAACTGTTTTAAAAAACGTTGATACACTCACTCCTTTGATTAAGAAATATGATGAGCCTATTCTGAAGCTCCTGACAGATATTAAAGTTAAGCTTTCAGATCCTGGCGAGCCCCTCAGTTTCACACTAGAATTTCACTTCAAACCCAATGAATATTTCAAAAATGAGTTGTTGACAAAGACCTATGTGCTGAAGTCAAAGCTAGCATATTATGATCCCCATCCCTATAGGGGAACTGCGATTGAGTATTCCACAGGCTGTGAGATAGATTGGAATGAAGGAAAGAATGTCACTTTGAAAACCATCAAGAAGAAACAGAAACATCGGATCTGGGGAACAATCCGAACTGTAACTGAAGATTTTCCCAAGGATTCATTTTTCAATTTTTTCTCTCCTCATGGAATCACCTCAAATGGAAGGGATGGAAATGATGATTTTTTACTTGGTCACAATTTACGTACTTACATAATTCCAAGATCAGTATTATTTTTCTCAGGTGATGCACTGGAATCTCAGCAGGAGGGGGTAGTTAGAGAAGTTAATGATGCAATTTATGACAAAATTATTTATGATAATTGGATGGCTGCAATTGAGGAAGTTAAAGCTTGTTGCAAAAACCTTGAGGCATTAGTAGAAGACATTGATCGTTAGAGCAGAGTATACATGGCCCTGAAATTAACTGCCCTAGATATAGTTACTCAAGGTATAAGAAGCCTTGTGTTCTGTATTTTTCTTGTAGTGTTAGTTAAAACATATGTTTCAAAAATATAAGAAAAGTTCAAAAACTAATTAATTTGACCTTGAGTTTTAGTAGTAGAATGTTTTCAAGAAATGTACACTGTGGTAAATGATTTAAAACACTAGTATAGTGTTGTGTAGCTTAATCCTTCTGAAGTCTTTTTGTCATGTAGCTATTAATCTGTGGCTATGAAATGATCAGAAATGCTAAGTGAGATCAATATTTGTTTGGAAAAAAAATCTTGGGAAACAACCCAAGGGTTTTCGCTGTTGTTGTTTTTCTTTTTCTATTTTTGTTTACTTAGTCCTTTAGCTAGTGGATTTAATTTTGTTGTGCCTGCTTCATTTTGCAATAACAATGCAGTAGAATTTAAAACTTGGATGCTTAAGAGGCCTGCATATAGATAAGAATTTCAGGCAAAACTACATTTATTGTTAATAACAGCTTGTTCATAGGCTCTTGTATTTTATGTAACTGTGATAAATAATGAAACTTAGTTATATTGAGGTTATTGTTTGTCGGTGAAGTGTTAGTCACAGTATTTTCAAAAGTTTGCACATATTGTTCTGTGTAATTGTGTAAGCCATAATTACAGTGTTTAATTCTCTTTTCCTATTACATCATTCATTGAAAGTGATCACTTTACCATTTTGAAAAGATATTTCGTGTTCTTTCACTGCAAAATAAAAAGAATAAAAATTTCAGAGTGTCTCATGTTTAAAA-3’

pGL3-NAP1L2-MUT（T marked in red is the mutation site）:

5’-AAGTGATAGCTGCGTAATCATACTGCGGCACCGTTTTTTTCTTGCAGCAGTAGCTGCT

TGCGGAGGAGGTCTGCCCACTGCAGCTCTCTGCAGTCTCCGGCTCTCTCCTGCAGGATCGGTCAACGCAGCCGTCGCCGCCCTCTGCACCCAGCCCAGGTCGCCACTGCTTCAGTCCGGTTCTCAAAGCCTCAGCACCATCTTTTATCCCCGAGCAGCCTGGATCGTCGTTCCCTCAGTCCGGACGCCACTGCTAGGTCCGACCACCGCCGCTTCTGATATTTCGGTGAGTCTTTTCCTGTGGAGGTTTGGTCTCCCGATCTCTGTGGTAGCCACCTTAGGCGTGTACGGTCCTTTGAAAAATGGCCGAGTCAGAGAACCGCAAGGAGCTGTCAGAATCCAGTCAAGAAGAGGCTGGTAATCAGATAATGGTGGAAGGGCTCGGGGAACATCTGGAGCGCGGTGAAGATGCCGCTGCTGGGCTTGGAGACGATGGGAAGTGCGGTGAAGAAGCTGCCGCTGGGCTTGGGGAAGAAGGGGAAAACGGTGAAGATACTGCTGCTGGGTCCGGGGAAGATGGGAAAAAAGGTGGCGATACTGATGAGGTCTCAGAGGCAGACCGTCCAAAAGGACTTATCGGTTATGTTTTAGATACAGACTTTGTTGAAAGTCTACCTGTGAAAGTTAAGTACCGTGTGTTAGCCCTTAAAAAGCTTCAAACTAGAGCGGCCAATTTAGAATCCAAATTCCTGAGGGAATTTCATGACATTGAAAGAAAGTTTGCTGAAATGTACCAACCCTTACTGGAAAAAAGACGTCAGATCATCAATGCAATCTATGAACCTACAGAAGAGGAATGTGAATATAAATCAGACTCTGAGGACTGTGATGATGAGGAAATGTGTCATGAAGAGATGTATGGTAATGAGGAGGGTATGGTACATGAATATGTGGATGAGGACGATGGTTATGAGGTCTATTATTATGATTATGCTGTGGAAGAGGAGGAGGAGGAGGAGGAGGAGGACGACATTGAGGCTACTGGAGAAGAGAATAAAGAAGAGGAGGATCCTAAGGGAATTCCTGATTTTTGGCTAACTGTTTTAAAAAACGTTGATACACTCACTCCTTTGATTAAGAAATATGATGAGCCTATTCTGAAGCTCCTGACAGATATTAAAGTTAAGCTTTCAGATCCTGGCGAGCCCCTCAGTTTCACACTAGAATTTCACTTCAAACCCAATGAATATTTCAAAAATGAGTTGTTGACAAAGACCTATGTGCTGAAGTCAAAGCTAGCATATTATGATCCCCATCCCTATAGGGGAACTGCGATTGAGTATTCCACAGGCTGTGAGATAGATTGGAATGAAGGAAAGAATGTCACTTTGAAAACCATCAAGAAGAAACAGAAACATCGGATCTGGGGATCAATCCGAACTGTAACTGAAGATTTTCCCAAGGATTCATTTTTCAATTTTTTCTCTCCTCATGGAATCACCTCAAATGGAAGGGATGGAAATGATGATTTTTTACTTGGTCACAATTTACGTACTTACATAATTCCAAGATCAGTATTATTTTTCTCAGGTGATGCACTGGAATCTCAGCAGGAGGGGGTAGTTAGAGAAGTTAATGATGCAATTTATGACAAAATTATTTATGATAATTGGATGGCTGCAATTGAGGAAGTTAAAGCTTGTTGCAAAAACCTTGAGGCATTAGTAGAAGACATTGATCGTTAGAGCAGAGTATACATGGCCCTGAAATTAACTGCCCTAGATATAGTTACTCAAGGTATAAGAAGCCTTGTGTTCTGTATTTTTCTTGTAGTGTTAGTTAAAACATATGTTTCAAAAATATAAGAAAAGTTCAAAAACTAATTAATTTGACCTTGAGTTTTAGTAGTAGAATGTTTTCAAGAAATGTACACTGTGGTAAATGATTTAAAACACTAGTATAGTGTTGTGTAGCTTAATCCTTCTGAAGTCTTTTTGTCATGTAGCTATTAATCTGTGGCTATGAAATGATCAGAAATGCTAAGTGAGATCAATATTTGTTTGGAAAAAAAATCTTGGGAAACAACCCAAGGGTTTTCGCTGTTGTTGTTTTTCTTTTTCTATTTTTGTTTACTTAGTCCTTTAGCTAGTGGATTTAATTTTGTTGTGCCTGCTTCATTTTGCAATAACAATGCAGTAGAATTTAAAACTTGGATGCTTAAGAGGCCTGCATATAGATAAGAATTTCAGGCAAAACTACATTTATTGTTAATAACAGCTTGTTCATAGGCTCTTGTATTTTATGTAACTGTGATAAATAATGAAACTTAGTTATATTGAGGTTATTGTTTGTCGGTGAAGTGTTAGTCACAGTATTTTCAAAAGTTTGCACATATTGTTCTGTGTAATTGTGTAAGCCATAATTACAGTGTTTAATTCTCTTTTCCTATTACATCATTCATTGAAAGTGATCACTTTACCATTTTGAAAAGATATTTCGTGTTCTTTCACTGCAAAATAAAAAGAATAAAAATTTCAGAGTGTCTCATGTTTAAAA-3’

pGL3-MMP2 promoter-WT（The yellow part indicates the core binding site of YY1 and the sense strand MMP2 promoter predicted by JASPAR; Filter: Relative profile score threshold>90%）：

5’-TTAAAAAAAGAAAACCAGAAAGTCAGATTTTTACATGAAGTTTCCCAAAT

TTCAAAATGCTGTTCAGGCTGGATTTAGCCCACAGGCCACGAGTTTGCAG

CCCCTGCTTTAGTGAGATAACTTTTTCCATTTTCACTCTCAGCTCTCAGC

TCTCCAACTTGGCTCTCTGGCTATCCACAGGACGTGGACATGAGCCCAGT

GGGGCTGGGCCAGGAGGCAATCCCCCTTCCCAACTGACCTCAGTCTCGCC

CTCTCCAAAACAGCCAAGGTTTGTCACTGGGTCAGGCTGAAGGGCCTGGC

TCCCTCCTGCGGGGCAAGGTCCCTCCCAAGAGGGTCCTTTAAAACTGACT

CTGGAAAGTCAGAGCACACACCCACCAGACAAGCCTGAACTTGTCTGAAG

CCCACTGAGACCCAAGCCGCAGAGACTTTTCTAGCTGTGATGATCAAGAC

ATAATCGTGACCTCCAATGCCCCCCACAAGTATATTGCTCCTGATTCTTT

CAGCCCCTGACCTTACTTCTCAAACTGTTCCCTGCTGACCCCCAGTCCTA

TCTGCCCCCTTCCTAGGCTGGTCCTTACTGACCCCTCCAGCTCCATCCCC

TCACCCTGTGCCCCACCTTTTTCAGATAGAAAAAACTTTCTTCTCCAGTG

CCTCTTGCTGTTTTTCATCTCTGGGCCATTGTCAATGTTCCCTAAAACAT

TCCCCATATTCCCCACCCAGCACTCCACCTCTTTAGCTCTTCAGGTCTCA

GCTCAGAAGTCACTTCTTCCAGGAAGCCTTCCTTGATTGTCTTTACTAGT

TTAGGGGCTGAAGTCAGGCGTTCCCAACAGCCTGCTGGAGTTCCCCATCA

CAGCTTATCTCTCAACTGTCTTTCCTGAGAGAGGGAGAAGACATTCCTCA

GAGACGGTTGTCACAGGGAGAACTTCAAAATTGGGATTCGACCTGAGAGG

CCACATGGATTCTTGGCTTGGCGCAGGAAAGGATTCAAGAGTGAGTGGGG

AATTCGTGGAACTGAGGGCTCCTCCCCTTTTTAGACCATATAGGGTAAAC

CTCCCCACATTGCCATGGCATTTATAAACTGCCATGGCACTGGTGGGTGC

TTCCTTTAACATGCTAATGCATTATAATTAGCGTAAAATGAGCAGTGAGG

ATGACCAGAGGTCGCTTTCTTTGCCATCTTGGTTTTGGCTGGCTTCTTCA

CTGCATACTGTTTTATCAGTGGGGTCTTTGTGACCTCTATCTTATTAAAC

CAGTCTTGCCCAATTTCTATCTCATCCTGTGACCGAGAATGCGGACCCTC

CTGGGAGTGCAGCCCAGCAGGTCTCAGCCTCATTTTACCCAGCCCCCTGT

TCAAGATGGAGTCGCTCTGGTTCCAACGTCTCTAACGCGGGGCCCCTGAC

TGCTCTATTTCCCAAGGTGTATCTAGCATCTCGCACTATACGAGGCCAAG

TTAAGGCTTACACATTTGCAGAAGGAAAGAGGTAAGGAAGCAACCTGGGA

CCTTCCACTGTCTCTGTTTCCATCTCTCTCTTTCCATCTCTGTTCATCCC

AGAATCTCTCTGTCCCTATCCCTAAATATCGAAAATTTCTGTCTCTGACC

ATCTATCATTGTGGCTGATCATCTGTTTCTGACCATTCCTTCCCGTTCCT

GACCCCAGGGAGTGCAGGGTGTCCTAGCCAAGCCGGCGTCCCTCCTAGTA

GTACCGCTGCTCTCTAACCTCAGGACGTCAAGGGCCTAGAGCGACAGATG

TTTCCCAGCAGGGGGTTCTGAGGCTGTGCGCCCAGATCGCGAGAGAGGCA

AGTGGGGTGACGAGGTCGTGCACTGAGGGTGGACGTAGAGGCCAGGAGTA

GCAGGCGGCCGGGGAAAAGAGGTGGAGAAAGGAAAAAAGAGGAGAAAAGT

GGAGGAGGGCGAGTAGGGGGGTGGGGCAGAGAGGGGCGGGCCCGAGTGCG

CCCCCCGCCCCCAGCCCCGCTCTGCCAGCTCCCTCCCAGCCCAGCCGGCT-3’

pGL3-MMP2 promoter-MUT (Mutation sites are marked in red.)：

5’-TTAAAAAAAGAAAACCAGAAAGTCAGATTTTTACATGAAGTTTCCCAAAT

TTCAAAATGCTGTTCAGGCTGGATTTAGCCCACAGGCCACGAGTTTGCAG

CCCCTGCTTTAGTGAGATAACTTTTTCCATTTTCACTCTCAGCTCTCAGC

TCTCCAACTTGGCTCTCTGGCTATCCACAGGACGTGGACATGAGCCCAGT

GGGGCTGGGCCAGGAGGCAATCCCCCTTCCCAACTGACCTCAGTCTCGCC

CTCTCCAAAACAGCCAAGGTTTGTCACTGGGTCAGGCTGAAGGGCCTGGC

TCCCTCCTGCGGGGCAAGGTCCCTCCCAAGAGGGTCCTTTAAAACTGACT

CTGGAAAGTCAGAGCACACACCCACCAGACAAGCCTGAACTTGTCTGAAG

CCCACTGAGACCCAAGCCGCAGAGACTTTTCTAGCTGTGATGATCAAGAC

ATAATCGTGACCTCCAATGCCCCCCACAAGTATATTGCTCCTGATTCTTT

CAGCCCCTGACCTTACTTCTCAAACTGTTCCCTGCTGACCCCCAGTCCTA

TCTGCCCCCTTCCTAGGCTGGTCCTTACTGACCCCTCCAGCTGGTACCCC

TCACCCTGTGCCCCACCTTTTTCAGATAGAAAAAACTTTCTTCTCCAGTG

CCTCTTGCTGTTTTTCATCTCTGGGGGTATGTCAATGTTCCCTAAAACAT

TCCCCATATTCCCCACCCAGCACTCCACCTCTTTAGCTCTTCAGGTCTCA

GCTCAGAAGTCACTTCTTCCAGGAAGCCTTCCTTGATTGTCTTTACTAGT

TTAGGGGCTGAAGTCAGGCGTTCCCAACAGCCTGCTGGAGTTCCCCATCA

CAGCTTATCTCTCAACTGTCTTTCCTGAGAGAGGGAGAAGACATTCCTCA

GAGACGGTTGTCACAGGGAGAACTTCAAAATTGGGATTCGACCTGAGAGG

CCACATGGATTCTTGGCTTGGCGCAGGAAAGGATTCAAGAGTGAGTGGGG

AATTCGTGGAACTGAGGGCTCCTCCCCTTTTTAGAGGTAATAGGGTAAAC

CTCCCCACATTGGGTAGGCATTTATAAACTGGGTAGGCACTGGTGGGTGC

TTCCTTTAACATGCTAATGCATTATAATTAGCGTAAAATGAGCAGTGAGG

ATGACCAGAGGTCGCTTTCTTTGGGTACTTGGTTTTGGCTGGCTTCTTCA

CTGCATACTGTTTTATCAGTGGGGTCTTTGTGACCTCTATCTTATTAAAC

CAGTCTTGCCCAATTTCTATCTCATCCTGTGACCGAGAATGCGGACCCTC

CTGGGAGTGCAGCCCAGCAGGTCTCAGCCTCATTTTACCCAGCCCCCTGT

TCAAGATGGAGTCGCTCTGGTTCCAACGTCTCTAACGCGGGGCCCCTGAC

TGCTCTATTTCCCAAGGTGTATCTAGCATCTCGCACTATACGAGGCCAAG

TTAAGGCTTACACATTTGCAGAAGGAAAGAGGTAAGGAAGCAACCTGGGA

CCTTCCACTGTCTCTGTTTGGTACTCTCTCTTTGGTACTCTGTTCATCCC

AGAATCTCTCTGTCCCTATCCCTAAATATCGAAAATTTCTGTCTCTGAGG

TACTATCATTGTGGCTGATCATCTGTTTCTGAGGTATCCTTCCCGTTCCT

GACCCCAGGGAGTGCAGGGTGTCCTAGCCAAGCCGGCGTCCCTCCTAGTA

GTACCGCTGCTCTCTAACCTCAGGACGTCAAGGGCCTAGAGCGACAGATG

TTTCCCAGCAGGGGGTTCTGAGGCTGTGCGCCCAGATCGCGAGAGAGGCA

AGTGGGGTGACGAGGTCGTGCACTGAGGGTGGACGTAGAGGCCAGGAGTA

GCAGGCGGCCGGGGAAAAGAGGTGGAGAAAGGAAAAAAGAGGAGAAAAGT

GGAGGAGGGCGAGTAGGGGGGTGGGGCAGAGAGGGGCGGGCCCGAGTGCG

CCCCCCGCCCCCAGCCCCGCTCTGCCAGCTCCCTCCCAGCCCAGCCGGCT-3’

pGL3-MMP9 promoter-WT（The yellow part indicates the core binding site of YY1 and the sense strand MMP2 promoter predicted by JASPAR; Filter: Relative profile score threshold>90%）：

5’- ACGGTGCTTGACACAGTAAATCTCAAAAAATGCATTATTATTATTATGGT

TCAGAGGTAAAGTGACTTGCCCAAGGTCACATAGCTGGAAAATGGCAGAG

CCGGGATGGAAATCCAGGACTTCGTGACTGCAAAGCAGATGTTCATTGGT

TAGTGAACTTTAGAACTTCAACTTTTCTGTAAAGGAAGTTAATTATCTCC

ATCTCACAGTCTCATTTATTAGATAAGCATATAAAATGCCTGGCACATAG

TAGGCCCTTTAAATACAGCTTATTGGGCCGGGCGCCATGGCTCATGCCCG

TAATCCTAGCACTTTGGGAGGCCAGGTGGGCAGATCACTTGAGTCAGAAG

TTCGAAACCAGCCTGGTCAACGTAGTGAAACCCCATCTCTACTAAAAATA

CAAAAAATTTAGCCAGGCGTGGTGGCGCACGCCTATAATACCAGCTACTC

GGGAGGCTGAGGCAGGAGAATTGCTTGAACCCGGGAGGCAGATGTTGCAG

TGAGCCGAGATCACGCCACTGCACTCCAGCCTGGGTGACAGAGTGATACT

ACACCCCCCAAAAATAAAATAAAATAAATAAATACAACTTTTTGAGTTGT

TAGCAGGTTTTTCCCAAATAGGGCTTTGAAGAAGGTGAATATAGACCCTG

CCCGATGCCGGCTGGCTAGGAAGAAAGGAGTGAGGGAGGCTGCTGGTGTG

GGAGGCTTGGGAGGGAGGCTTGGCATAAGTGTGATAATTGGGGCTGGAGA

TTTGGCTGCATGGAGCAGGGCTGGAGAACTGAAAGGGCTCCTATAGATTA

TTTTCCCCCATATCCTGCCCCAATTTGCAGTTGAAGAATCCTAAGCTGAC

AAAGGGGAAGGCATTTACTCCAGGTTACACTGCAGCTTAGAGCCCAATAA

CCTGGTTTGGTGATTCCAAGTTAGAATCATGGTCTTTTGGCAGGGTCTCG

CTCTGTTGCCCAGGCTGGAGTGCAGTGACATAATCATGGCTCACTGTATC

CTTGACCTTCTTTCTGGGCTCAAGCAATCCTCCCACCTCGGCCTCCCAAA

GTGCTAAGATTACAGGAATGAGCCACCATACCTGGCCCTGAATCTTGGGT

CTTGGCCTTAGTAATTAAAACCAATCACCACCATCCGTTGCGGACTTACA

ACCTACAGTGTTCTAAACATTTTATATGTTTGATCTCATTTAATCCTCAC

ATCAATTTAGGGACAAAGAGCCCCCCACCCCCCGTTTTTTTTTTTACAGC

TGAGGAAACACTTCAAAGTGGTAAGACATTTGCCCGAGGTCCTGAAGGAA

GAGAGTAAAGCCATGTCTGCTGTTTTCTAGAGGCTGCTACTGTCCCCTTT

ACTGCCCTGAAGATTCAGCCTGCGGAAGACAGGGGGTTGCCCCAGTGGAA

TTCCCCAGCCTTGCCTAGCAGAGCCCATTCCTTCCGCCCCCAGATGAAGC

AGGGAGAGGAAGCTGAGTCAAAGAAGGCTGTCAGGGAGGGAAAAAGAGGA

CAGAGCCTGGAGTGTGGGGAGGGGTTTGGGGAGGATATCTGACCTGGGAG

GGGGTGTTGCAAAAGGCCAAGGATGGGCCAGGGGGATCATTAGTTTCAGA

AAGAAGTCTCAGGGAGTCTTCCATCACTTTCCCTTGGCTGACCACTGGAG

GCTTTCAGACCAAGGGATGGGGGATCCCTCCAGCTTCATCCCCCTCCCTC

CCTTTCATACAGTTCCCACAAGCTCTGCAGTTTGCAAAACCCTACCCCTC

CCCTGAGGGCCTGCGGTTTCCTGCGGGTCTGGGGTCTTGCCTGACTTGGC

AGTGGAGACTGCGGGCAGTGGAGAGAGGAGGAGGTGGTGTAAGCCCTTTC

TCATGCTGGTGCTGCCACACACACACACACACACACACACACACACACAC

ACACACACACACCCTGACCCCTGAGTCAGCACTTGCCTGTCAAGGAGGGG

TGGGGTCACAGGAGCGCCTCCTTAAAGCCCCCACAACAGCAGCTGCAGTC-3’

pGL3-MMP9 promoter-MUT (Mutation sites are marked in red.):

5’- ACGGTGCTTGACACAGTAAATCTCAAAAAATGCATTATTATTATTATGGT

TCAGAGGTAAAGTGACTTGCCCAAGGTCACATAGCTGGAAAATGGCAGAG

CCGGGATGGAAATCCAGGACTTCGTGACTGCAAAGCAGATGTTCATTGGT

TAGTGAACTTTAGAACTTCAACTTTTCTGTAAAGGAAGTTAATTATCTGG

TACTCACAGTCTCATTTATTAGATAAGCATATAAAATGCCTGGCACATAG

TAGGCCCTTTAAATACAGCTTATTGGGCCGGGCGGGTAGGCTCATGCCCG

TAATCCTAGCACTTTGGGAGGCCAGGTGGGCAGATCACTTGAGTCAGAAG

TTCGAAACCAGCCTGGTCAACGTAGTGAAACCCCATCTCTACTAAAAATA

CAAAAAATTTAGCCAGGCGTGGTGGCGCACGCCTATAATACCAGCTACTC

GGGAGGCTGAGGCAGGAGAATTGCTTGAACCCGGGAGGCAGATGTTGCAG

TGAGCCGAGATCACGCCACTGCACTCCAGCCTGGGTGACAGAGTGATACT

ACACCCCCCAAAAATAAAATAAAATAAATAAATACAACTTTTTGAGTTGT

TAGCAGGTTTTTCCCAAATAGGGCTTTGAAGAAGGTGAATATAGACCCTG

CCCGATGCCGGCTGGCTAGGAAGAAAGGAGTGAGGGAGGCTGCTGGTGTG

GGAGGCTTGGGAGGGAGGCTTGGCATAAGTGTGATAATTGGGGCTGGAGA

TTTGGCTGCATGGAGCAGGGCTGGAGAACTGAAAGGGCTCCTATAGATTA

TTTTCCCCCATATCCTGCCCCAATTTGCAGTTGAAGAATCCTAAGCTGAC

AAAGGGGAAGGCATTTACTCCAGGTTACACTGCAGCTTAGAGCCCAATAA

CCTGGTTTGGTGATTCCAAGTTAGAATCATGGTCTTTTGGCAGGGTCTCG

CTCTGTTGCCCAGGCTGGAGTGCAGTGACATAATCATGGCTCACTGTATC

CTTGACCTTCTTTCTGGGCTCAAGCAATCCTCCCACCTCGGCCTCCCAAA

GTGCTAAGATTACAGGAATGAGCCAGGTAACCTGGCCCTGAATCTTGGGT

CTTGGCCTTAGTAATTAAAACCAATCACCAGGTACCGTTGCGGACTTACA

ACCTACAGTGTTCTAAACATTTTATATGTTTGATCTCATTTAATCCTCAC

ATCAATTTAGGGACAAAGAGCCCCCCACCCCCCGTTTTTTTTTTTACAGC

TGAGGAAACACTTCAAAGTGGTAAGACATTTGCCCGAGGTCCTGAAGGAA

GAGAGTAAAGGGTAGTCTGCTGTTTTCTAGAGGCTGCTACTGTCCCCTTT

ACTGCCCTGAAGATTCAGCCTGCGGAAGACAGGGGGTTGCCCCAGTGGAA

TTCCCCAGCCTTGCCTAGCAGAGCCCATTCCTTCCGCCCCCAGATGAAGC

AGGGAGAGGAAGCTGAGTCAAAGAAGGCTGTCAGGGAGGGAAAAAGAGGA

CAGAGCCTGGAGTGTGGGGAGGGGTTTGGGGAGGATATCTGACCTGGGAG

GGGGTGTTGCAAAAGGCCAAGGATGGGCCAGGGGGATCATTAGTTTCAGA

AAGAAGTCTCAGGGAGTCTTGGTACACTTTCCCTTGGCTGACCACTGGAG

GCTTTCAGACCAAGGGATGGGGGATCCCTCCAGCTTCATCCCCCTCCCTC

CCTTTCATACAGTTCCCACAAGCTCTGCAGTTTGCAAAACCCTACCCCTC

CCCTGAGGGCCTGCGGTTTCCTGCGGGTCTGGGGTCTTGCCTGACTTGGC

AGTGGAGACTGCGGGCAGTGGAGAGAGGAGGAGGTGGTGTAAGCCCTTTC

TCATGCTGGTGCTGCCACACACACACACACACACACACACACACACACAC

ACACACACACACCCTGACCCCTGAGTCAGCACTTGCCTGTCAAGGAGGGG

TGGGGTCACAGGAGCGCCTCCTTAAAGCCCCCACAACAGCAGCTGCAGTC-3’
