# Supplementary material for: LncNAP1L6 activates MMP pathway by stabilizing the m6A-modified NAP1L2 to promote malignant progression in prostate cancer
Source: Cancer Gene Ther. 2022 Oct 4;30(1):209–18. doi: 10.1038/s41417-022-00537-3 (PMC9842505; doi:10.1038/s41417-022-00537-3)
Supplement: Supplementary file 2 [file 41417_2022_537_MOESM2_ESM.docx]

shRNA sequence:

sh-NC:

5’-CCGGAAGATTATCTTGACACACTTACTCGAGTAAGTGTGTCAAGATAATCTTTTTTTG-3’

sh-lncNAP1L6-1:

5’-CCGGAGTAATGGGCCCATTTCACCGCTCGAGCGGTGAAATGGGCCCATTACTTTTTTG-3’

sh-lncNAP1L6-2:

5’-CCGGTCGATTTCCTGAACTTGTGATCTCGAGATCACAAGTTCAGGAAATCGATTTTTG-3’

sh-lncNAP1L6-3:

5’-CCGGTCATAAACCGGATTATTACATCTCGAGATGTAATAATCCGGTTTATGATTTTTG-3’

sh-NC:

5’-CCGGCTTCGGGGATATCCTATATGTCTCGAGACATATAGGATATCCCCGAAGTTTTTG-3’

sh-METTL14-1：

5’-CCGGGCTTAACCCATTAGTACTATCCTCGAGGATAGTACTAATGGGTTAAGCTTTTTG-3’

sh-METTL14-2：

5’-CCGGGCTGCTTTGTCGGTTGAAAGTCTCGAGACTTTCAACCGACAAAGCAGCTTTTTG-3’

sh-METTL14-3：

5’-CCGGGCTTATGCAGAAGGTCTTTCTCTCGAGAGAAAGACCTTCTGCATAAGCTTTTTG-3’

sh-NC：

5’-CCGGTACGTCTACCAAACCAACTCGCTCGAGCGAGTTGGTTTGGTAGACGTATTTTTG-3’

sh-METTL3-1：

5’-CCGGGCGTGAGAATTGGCTATATCCCTCGAGGGATATAGCCAATTCTCACGCTTTTTG-3’

sh-METTL3-2：

5’-CCGGGCAAGAATTCTGTGACTATGGCTCGAGCCATAGTCACAGAATTCTTGCTTTTTG-3’

sh-METTL3-3：

5’-CCGGGCTCAACATACCCGTACTACACTCGAGTGTAGTACGGGTATGTTGAGCTTTTTG-3’

sh-NC：

5’-CCGGCCGGACATTGTGGTAGGATTACTCGAGTAATCCTACCACAATGTCCGGTTTTTG-3’

sh-HNRNPC-1：

5’-CCGGGCATGTTGCATTGAGGAGTCACTCGAGTGACTCCTCAATGCAACATGCTTTTTG-3’

sh-HNRNPC-2：

5’-CCGGGGCACCCTTTCTGAGGTTTGTCTCGAGACAAACCTCAGAAAGGGTGCCTTTTTG-3’

sh-HNRNPC-3：

5’-CCGGGCACAGAGCAGTCTGTTTAGCCTCGAGGCTAAACAGACTGCTCTGTGCTTTTTG-3’

sh-NC：

5’-CCGGCCGTGCTGTACTGATTATCAACTCGAGTTGATAATCAGTACAGCACGGTTTTTG-3’

sh-NAP1L2-1：

5’-CCGGTGTATCTAAAACATAACCGATCTCGAGATCGGTTATGTTTTAGATACATTTTTG-3’

sh-NAP1L2-2：

5’-CCGGATTTGGATTCTAAATTGGCCGCTCGAGCGGCCAATTTAGAATCCAAATTTTTTG-3’

sh-NAP1L2-3：

5’-CCGGTTTGACTTCAGCACATAGGTCCTCGAGGACCTATGTGCTGAAGTCAAATTTTTG-3’

sh-NC：

5’-CCGGGGTTTTACTTTGTGTGGTGGTCTCGAGACCACCACACAAAGTAAAACCTTTTTG-3’

sh-YY1-1：

5’-CCGGGCTCCAAGAACAATAGCTTGCCTCGAGGCAAGCTATTGTTCTTGGAGCTTTTTG-3’

sh-YY1-2：

5’-CCGGGCAAGTGTGAGTGAAGCATCTCTCGAGAGATGCTTCACTCACACTTGCTTTTTG-3’

sh-YY1-3：

5’-CCGGGGTCGTTGGTTATTTGGTTTGCTCGAGCAAACCAAATAACCAACGACCTTTTTG-3’
