# Supplementary material for: LncNAP1L6 activates MMP pathway by stabilizing the m6A-modified NAP1L2 to promote malignant progression in prostate cancer
Source: Cancer Gene Ther. 2022 Oct 4;30(1):209–18. doi: 10.1038/s41417-022-00537-3 (PMC9842505; doi:10.1038/s41417-022-00537-3)
Supplement: Supplementary file 3 — Supplementary Legends [file 41417_2022_537_MOESM3_ESM.docx]

**Supplementary Figures Legends**

**LncNAP1L6 activates MMP pathway by stabilizing the m6A-modified NAP1L2 to promote malignant progression in prostate cancer**

Yuxiao Zheng^1*^, Feng Qi^1*^, Lu Li^2, 3*^, Bin Yu^1*^, Yifei Cheng^4^, Minghui Ge^2, 3^, Chao Qin^5#^, Xiao Li^1,6#^

1. Department of Urologic Surgery, Jiangsu Cancer Hospital & Jiangsu Institute of Cancer Research & Affiliated Cancer Hospital of Nanjing Medical University, Nanjing, China.
2. State Key Laboratory of Translational Medicine and Innovative Drug Development, Jiangsu Simcere Diagnostics Co., Ltd., Nanjing, China.
3. Nanjing Simcere Medical Laboratory Science Co., Ltd., Nanjing, China.
4. Department of Urology, The First Affiliated Hospital of Nanjing Medical University, Nanjing, China.
5. State Key Laboratory of Reproductive Medicine, Department of Urology, The First Affiliated Hospital of Nanjing Medical University, Nanjing, China.
6. Department of Scientific Research, Jiangsu Cancer Hospital & Jiangsu Institute of Cancer Research & Affiliated Cancer Hospital of Nanjing Medical University, Nanjing, China.

***Yuxiao Zheng, Feng Qi, Lu Li and Bin Yu contributed equally to this work.**

**#Corresponding author:**

**Dr. Xiao Li**

**Address**:

Department of Urologic Surgery, Department of Scientific Research, Jiangsu Cancer Hospital & Jiangsu Institute of Cancer Research & Affiliated Cancer Hospital of Nanjing Medical University, Nanjing 210009, China.

1. **mail**: [leex91@163.com](mailto:leex91@163.com)

**Dr. Chao Qin**

**Address**: State Key Laboratory of Reproductive Medicine, Department of Urology, The First Affiliated Hospital of Nanjing Medical University, Nanjing 210029, China.

**E-mail**: qinchao@njmu.edu.cn

**Figure S1**: Supplementary Figure. (Fig.S1 A) The association between METTL3 and NAP1L2 expression. (Fig.S1 B) The evidence that METTL14 is positively associated with NAP1L2. The data are derived from the analysis of PRAD tissue in the GEPIA database. (Fig.S1 C) The total m6A level in PC3 cells was significantly reduced after METTL3 interference. (Fig.S1 D) The m6A level of NAP1L2 mRNA was reduced after METTL3 interference.
