# Supplementary material for: LncNAP1L6 activates MMP pathway by stabilizing the m6A-modified NAP1L2 to promote malignant progression in prostate cancer
Source: Cancer Gene Ther. 2022 Oct 4;30(1):209–18. doi: 10.1038/s41417-022-00537-3 (PMC9842505; doi:10.1038/s41417-022-00537-3)
Supplement: Supplementary file 4 — Supplementary tables [file 41417_2022_537_MOESM4_ESM.docx]

**Supplementary table legends:**

**Table S1.** Co-expression genes of NAP1L2 in PCa.

| **Gene Symbol** | **Gene ID** | **PCC** |
| --- | --- | --- |
| RRM2B | ENSG00000048392.11 | 0.66 |
| SELT | ENSG00000198843.12 | 0.63 |
| OSBP | ENSG00000110048.11 | 0.62 |
| NDFIP1 | ENSG00000131507.10 | 0.62 |
| TMEM168 | ENSG00000146802.12 | 0.61 |
| UBE2W | ENSG00000104343.19 | 0.6 |
| RAB6A | ENSG00000175582.19 | 0.6 |
| TERF1 | ENSG00000147601.13 | 0.6 |
| YY1 | ENSG00000100811.10 | 0.6 |
| ATP6V1C1 | ENSG00000155097.11 | 0.6 |
| ENPP4 | ENSG00000001561.6 | 0.59 |
| BBS10 | ENSG00000179941.6 | 0.59 |
| RAB14 | ENSG00000119396.10 | 0.59 |
| TTC8 | ENSG00000165533.18 | 0.59 |
| ATP7A | ENSG00000165240.17 | 0.59 |
| PAIP2 | ENSG00000120727.12 | 0.59 |
| METTL6 | ENSG00000206562.11 | 0.59 |
| RPS6KA5 | ENSG00000100784.9 | 0.59 |
| KIAA1143 | ENSG00000163807.5 | 0.59 |
| YME1L1 | ENSG00000136758.18 | 0.59 |

**Table S2.** The potential binding sites between YY1 and MMP2.

| **#MMP2** |  |  |  |  |  |  |  |  |
| --- | --- | --- | --- | --- | --- | --- | --- | --- |
| **Matrix ID** | **Name** | **Score** | **Relative score** | **Sequence ID** | **Start** | **End** | **Strand** | **Predicted sequence** |
| MA0095.1 | MA0095.1.YY1 | 8.38313 | 1 | NC_000016.10:55476830-55478929 | 1512 | 1517 | + | GCCATC |
| MA0095.1 | MA0095.1.YY1 | 8.191275 | 0.990201 | NC_000016.10:55476830-55478929 | 1937 | 1942 | + | ACCATC |
| MA0095.1 | MA0095.1.YY1 | 7.388649 | 0.949208 | NC_000016.10:55476830-55478929 | 931 | 936 | + | TCCATC |
| MA0095.1 | MA0095.1.YY1 | 7.388649 | 0.949208 | NC_000016.10:55476830-55478929 | 1694 | 1699 | - | TCCATC |
| MA0095.1 | MA0095.1.YY1 | 7.388649 | 0.949208 | NC_000016.10:55476830-55478929 | 1858 | 1863 | + | TCCATC |
| MA0095.1 | MA0095.1.YY1 | 7.388649 | 0.949208 | NC_000016.10:55476830-55478929 | 1872 | 1877 | + | TCCATC |
| MA0095.1 | MA0095.1.YY1 | 7.219338 | 0.940561 | NC_000016.10:55476830-55478929 | 291 | 296 | + | GCCATA |
| MA0095.1 | MA0095.1.YY1 | 7.219338 | 0.940561 | NC_000016.10:55476830-55478929 | 1014 | 1019 | + | GCCATT |
| MA0095.1 | MA0095.1.YY1 | 7.219338 | 0.940561 | NC_000016.10:55476830-55478929 | 1401 | 1406 | + | GCCATG |
| MA0095.1 | MA0095.1.YY1 | 7.219338 | 0.940561 | NC_000016.10:55476830-55478929 | 1403 | 1408 | - | GCCATG |
| MA0095.1 | MA0095.1.YY1 | 7.219338 | 0.940561 | NC_000016.10:55476830-55478929 | 1420 | 1425 | + | GCCATG |
| MA0095.1 | MA0095.1.YY1 | 7.219338 | 0.940561 | NC_000016.10:55476830-55478929 | 1422 | 1427 | - | GCCATG |
| MA0095.1 | MA0095.1.YY1 | 7.027483 | 0.930762 | NC_000016.10:55476830-55478929 | 1374 | 1379 | + | ACCATA |
| MA0095.1 | MA0095.1.YY1 | 7.027483 | 0.930762 | NC_000016.10:55476830-55478929 | 1971 | 1976 | + | ACCATT |
| MA0095.1 | MA0095.1.YY1 | 6.399622 | 0.898695 | NC_000016.10:55476830-55478929 | 1183 | 1188 | + | CCCATC |
| MA0095.1 | MA0095.1.YY1 | 6.224856 | 0.889769 | NC_000016.10:55476830-55478929 | 295 | 300 | - | TCCATA |
| MA0095.1 | MA0095.1.YY1 | 6.224856 | 0.889769 | NC_000016.10:55476830-55478929 | 465 | 470 | + | TCCATT |
| MA0095.1 | MA0095.1.YY1 | 6.224856 | 0.889769 | NC_000016.10:55476830-55478929 | 1293 | 1298 | - | TCCATG |
| MA0095.1 | MA0095.1.YY1 | 5.315089 | 0.843304 | NC_000016.10:55476830-55478929 | 1489 | 1494 | - | GTCATC |
| MA0095.1 | MA0095.1.YY1 | 5.23583 | 0.839256 | NC_000016.10:55476830-55478929 | 1042 | 1047 | + | CCCATA |
| MA0095.1 | MA0095.1.YY1 | 5.123234 | 0.833506 | NC_000016.10:55476830-55478929 | 778 | 783 | - | ATCATC |
| MA0095.1 | MA0095.1.YY1 | 5.123234 | 0.833506 | NC_000016.10:55476830-55478929 | 1957 | 1962 | + | ATCATC |

**Table S3.** The potential binding sites between YY1 and MMP9.

| **#MMP9** |  |  |  |  |  |  |  |  |
| --- | --- | --- | --- | --- | --- | --- | --- | --- |
| **Matrix ID** | **Name** | **Score** | **Relative score** | **Sequence ID** | **Start** | **End** | **Strand** | **Predicted sequence** |
| MA0095.1 | MA0095.1.YY1 | 8.191275 | 0.990201 | NC_000020.11:46006908-46009007 | 1130 | 1135 | + | ACCATC |
| MA0095.1 | MA0095.1.YY1 | 7.388649 | 0.949208 | NC_000020.11:46006908-46009007 | 105 | 110 | - | TCCATC |
| MA0095.1 | MA0095.1.YY1 | 7.388649 | 0.949208 | NC_000020.11:46006908-46009007 | 198 | 203 | + | TCCATC |
| MA0095.1 | MA0095.1.YY1 | 7.388649 | 0.949208 | NC_000020.11:46006908-46009007 | 1620 | 1625 | + | TCCATC |
| MA0095.1 | MA0095.1.YY1 | 7.219338 | 0.940561 | NC_000020.11:46006908-46009007 | 91 | 96 | - | GCCATT |
| MA0095.1 | MA0095.1.YY1 | 7.219338 | 0.940561 | NC_000020.11:46006908-46009007 | 284 | 289 | + | GCCATG |
| MA0095.1 | MA0095.1.YY1 | 7.219338 | 0.940561 | NC_000020.11:46006908-46009007 | 286 | 291 | - | GCCATG |
| MA0095.1 | MA0095.1.YY1 | 7.219338 | 0.940561 | NC_000020.11:46006908-46009007 | 985 | 990 | - | GCCATG |
| MA0095.1 | MA0095.1.YY1 | 7.219338 | 0.940561 | NC_000020.11:46006908-46009007 | 1310 | 1315 | + | GCCATG |
| MA0095.1 | MA0095.1.YY1 | 7.027483 | 0.930762 | NC_000020.11:46006908-46009007 | 45 | 50 | - | ACCATA |
| MA0095.1 | MA0095.1.YY1 | 7.027483 | 0.930762 | NC_000020.11:46006908-46009007 | 928 | 933 | - | ACCATG |
| MA0095.1 | MA0095.1.YY1 | 7.027483 | 0.930762 | NC_000020.11:46006908-46009007 | 1075 | 1080 | + | ACCATA |
| MA0095.1 | MA0095.1.YY1 | 7.027483 | 0.930762 | NC_000020.11:46006908-46009007 | 2017 | 2022 | + | ACCATG |
| MA0095.1 | MA0095.1.YY1 | 6.399622 | 0.898695 | NC_000020.11:46006908-46009007 | 382 | 387 | + | CCCATC |
| MA0095.1 | MA0095.1.YY1 | 6.399622 | 0.898695 | NC_000020.11:46006908-46009007 | 1572 | 1577 | - | CCCATC |
| MA0095.1 | MA0095.1.YY1 | 6.399622 | 0.898695 | NC_000020.11:46006908-46009007 | 1666 | 1671 | - | CCCATC |
| MA0095.1 | MA0095.1.YY1 | 6.224856 | 0.889769 | NC_000020.11:46006908-46009007 | 759 | 764 | - | TCCATG |
| MA0095.1 | MA0095.1.YY1 | 5.23583 | 0.839256 | NC_000020.11:46006908-46009007 | 807 | 812 | + | CCCATA |
| MA0095.1 | MA0095.1.YY1 | 5.23583 | 0.839256 | NC_000020.11:46006908-46009007 | 1424 | 1429 | + | CCCATT |
