# Supplementary figures and images for: LncNAP1L6 activates MMP pathway by stabilizing the m6A-modified NAP1L2 to promote malignant progression in prostate cancer
Source: Cancer Gene Ther. 2022 Oct 4;30(1):209–18. doi: 10.1038/s41417-022-00537-3 (PMC9842505; doi:10.1038/s41417-022-00537-3)

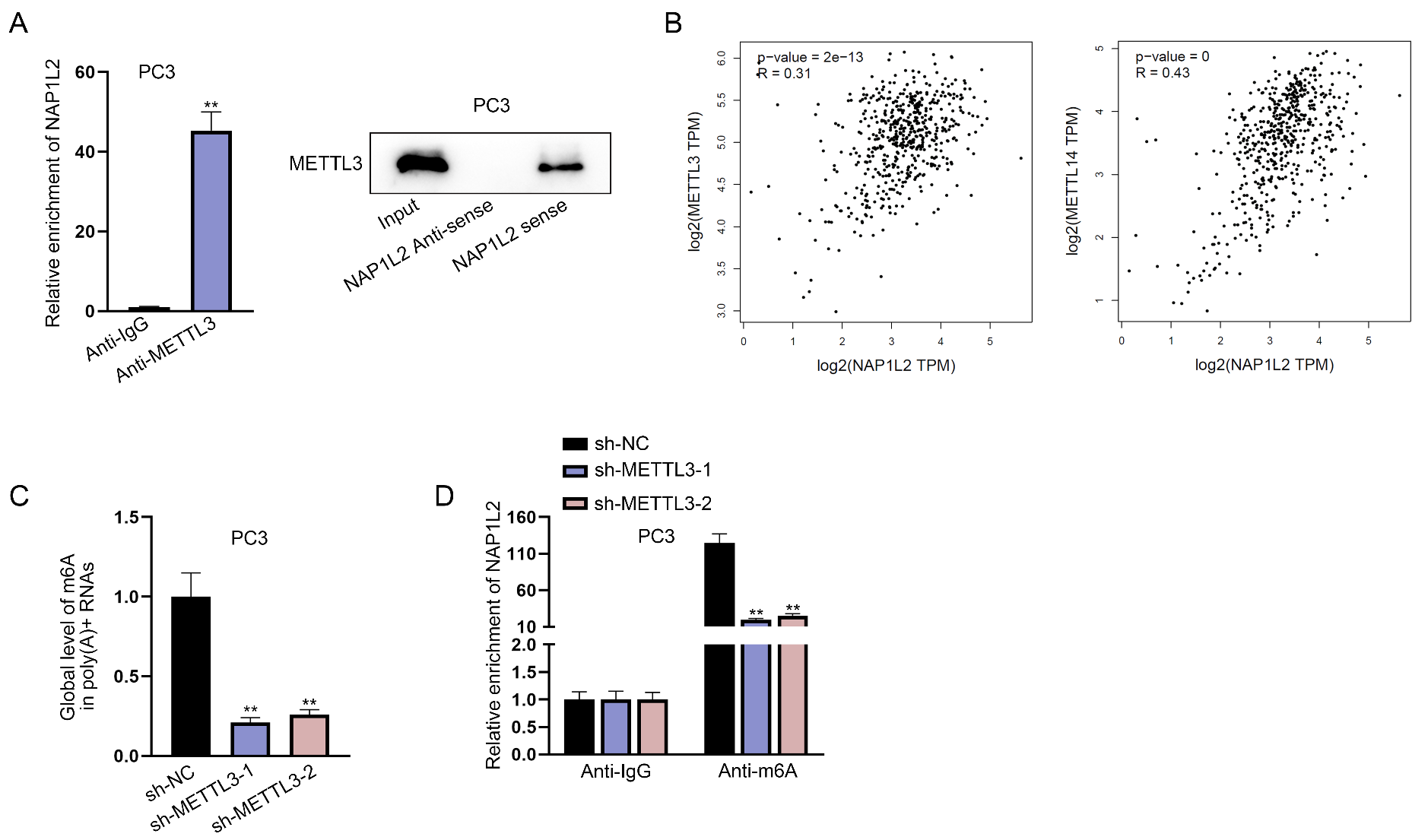

Supplement: Supplementary file 5 — Figure S1 [file 41417_2022_537_MOESM5_ESM.tif]
